# Imaging the Efficiency of Poly(3,4‐ethylenedioxythiophene) Doped with Acid‐Functionalized Carbon Nanotube and Iridium Oxide Electrode Coatings for Microstimulation

**DOI:** 10.1002/anbr.202000092

**Published:** 2021-05-03

**Authors:** Xin S. Zheng, Qianru Yang, Alberto L. Vazquez, Xinyan Tracy Cui

**Affiliations:** ^1^ Department of Bioengineering University of Pittsburgh 3501 Fifth Ave. Pittsburgh PA 15213 USA; ^2^ Departments of Radiology and Bioengineering University of Pittsburgh 3025 E. Carson St. Pittsburgh PA 15203 USA

**Keywords:** conducting polymers, electrical stimulation efficiencies, iridium oxide, mesoscale imaging, two-photon microscopy

## Abstract

Electrical microstimulation has shown promise in restoring neural deficits in humans. Electrodes coated with materials like the conducting polymer poly(3,4‐ethylenedioxythiophene) doped with acid‐functionalized carbon nanotubes (PEDOT/CNTs, or PC) exhibit superior charge injection than traditional metals like platinum. However, the stimulation performance of PC remains to be fully characterized. Advanced imaging techniques and transgenic tools allow for real‐time observations of neural activity in vivo. Herein, microelectrodes coated with PC and iridium oxide (IrOx) (a commonly used high‐charge‐injection material) are implanted in GCaMP6s mice and electrical stimulation is applied while imaging neuronal calcium responses. Results show that PC‐coated electrodes stimulate more intense and broader GCaMP responses than IrOx. Two‐photon microscopy reveals that PC‐coated electrodes activate significantly more neuronal soma and neuropil than IrOx‐coated electrodes in constant‐voltage stimulation and significantly more neuronal soma in constant‐current stimulation. Furthermore, with the same injected charge, both materials activate more spatially confined neural elements with shorter pulses than longer pulses, providing a means to tune stimulation selectivity. Finite element analyses reveal that the PC coating creates a denser and nonuniform electric field, increasing the likelihood of activating nearby neural elements. PC coating can significantly improve energy efficiency for electrical stimulation applications.

## Graphical abstract

The stimulation performance of two high‐charge‐injection materials poly(3,4‐ethylenedioxythiophene) doped with acid‐functionalized carbon nanotube (PEDOT/CNT) and iridium oxide is examined. In vivo imaging results show that PEDOT/CNT is a more energy‐efficient material for cortical activation. In addition, for both materials, shorter stimulation pulses activate more spatially distinct neural elements than longer pulses, providing a means to tune stimulation selectivity.

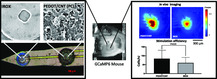

## 1 Introduction

Electrical microstimulation is a technique to activate a small population of neurons by passing currents through a microelectrode. In neuroscience research, microstimulation is used to study neural circuits, evaluate connected networks, and modulate behaviors.^[^ ^1^, ^2^, ^3^ ^]^ In humans, electrical microstimulation has been used to partially restore vision,^[^ ^4^, ^5^, ^6^, ^7^, ^8^, ^9^ ^]^ hearing,^[^ ^10^ ^]^ movement,^[^ ^11^ ^]^ and sensation.^[^ ^12^, ^13^ ^]^ Moreover, electrical stimulation, or bioelectronic medicine, has become an emerging alternative or complement to costly biologic drugs for the treatment of health issues such as arthritis,^[^ ^14^ ^]^ asthma,^[^ ^15^ ^]^ diabetes,^[^ ^16^ ^]^ and digestive disorders.^[^ ^17^ ^]^

Traditionally, microelectrodes are made up of metals with geometric surface areas between 2000 and 10 000 μm^2^,^[^ ^18^ ^]^ which are much smaller than macroelectrodes used for deep brain stimulation.^[^ ^19^ ^]^ This small electrode size offers increased spatial selectivity of the neural tissue but requires higher charge density which often exceeds charge injection limits (CILs) for safe stimulation. Efficient and safe electrical stimulation requires sufficient charge without exceeding potentials for irreversible chemical reactions.^[^ ^18^ ^]^ Platinum (Pt) is widely used for auditory^[^ ^20^ ^]^ and visual prostheses.^[^ ^9^ ^]^ However, new generations of auditory and visual prostheses are requiring higher channel counts with smaller electrodes for increased spatial selectivity and the CIL of conventional Pt electrodes is below the threshold for activation of these applications. Therefore, there is a critical need for higher CIL electrode materials. Examples of such materials are iridium oxide (IrOx),^[^ ^21^, ^22^, ^23^, ^24^ ^]^ titanium nitride (TiN),^[^ ^25^ ^]^ glassy carbon,^[^ ^26^ ^]^ and nanostructured Pt.^[^ ^27^ ^]^ These materials have shown dramatic improvement in CIL, on the order of mC cm^−2^, higher than smooth Pt (35–100 μC cm^−2^) in vitro, due to the increased electrochemical surface area. Particularly, IrOx has become more prevalent for neural stimulation electrodes in a variety of animal and human stimulation studies.^[^ ^28^, ^29^ ^]^ However, the high CIL of IrOx has been reported to decrease substantially over time and long‐lasting high CIL stimulation (for over 7 h) has led to IrOx degradation with adjacent neuronal degeneration,^[^ ^30^ ^]^ highlighting the importance of developing and evaluating new materials for electrical microstimulation. Conducting polymers (CP) such as poly(3,4‐ethylenedioxythiophene) (PEDOT) are polymers with a conjugated backbone of alternating double and single bonds. Electrical conductivity is achieved by doping the polymer with negatively charged ions (counter ions or dopants). By coating CPs onto metal substrates, we have drastically reduced electrical impedance and improved neurophysiological recording capabilities.^[^ ^31^, ^32^, ^33^, ^34^, ^35^, ^36^ ^]^ The increased electrochemical surface area and decreased impedance make PEDOT an ideal candidate for electrical stimulation.^[^ ^37^, ^38^, ^39^, ^40^, ^41^, ^42^ ^]^ Functionalized carbon nanotubes (CNTs) have been increasingly popular for their intriguing properties as a dopant for PEDOT.^[^ ^33^, ^43^, ^44^, ^45^, ^46^, ^47^ ^]^ The incorporation of CNTs not only significantly increases the electrochemical surface area thereby increasing the electrical conductivity of the electrode surface, but also improves the mechanical and electrochemical stability of the PEDOT coating during prolonged stimulation.^[^ ^43^, ^44^ ^]^ For most of these emerging stimulation materials, comprehensive evaluation of their microstimulation efficiency, safety, and longevity in vivo remain to be completed.

Examining the effects of electrical stimulation in vivo has been limited to electrophysiology,^[^ ^45^, ^48^, ^49^ ^]^ behavior,^[^ ^50^, ^51^, ^52^, ^53^ ^]^ and endpoint histology.^[^ ^53^, ^54^, ^55^, ^56^ ^]^ While these robust methods provide users with a functional and histological understanding of electrical stimuli, they do not provide direct visualization of the cellular response in real time. Recent advancements in vivo imaging techniques, such as mesoscale fluorescence microscopy and two‐photon microscopy (TPM), coupled with newly developed genetically engineered rodent models expressing fluorescent calcium indicators allow for the direct visualization of neuronal activity in response to electrode implantation and stimulation in vivo.^[^ ^57^, ^58^ ^]^ Using in vivo two‐photon calcium imaging in mice, rats, and cat models, Histed et al. revealed a sparse, distributed population of cortical neurons by electrical microstimulation via glass pipettes containing tungsten and platinum–iridium microwire electrodes.^[^ ^59^ ^]^ In more recent studies, the Kozai and coworkers utilized mesoscale fluorescence microscopy and two‐photon imaging to investigate the calcium responses to prolonged electrical stimulation in Thy1‐GCaMP6s mice and reported the effect of stimulation frequency,^[^ ^60^ ^]^ pulse symmetry, and phase order,^[^ ^61^ ^]^ using Michigan planar arrays.^[^ ^62^ ^]^ These studies demonstrate the capability of fluorescence imaging to characterize the neuronal response to stimulation at cellular and mesoscopic spatial scales in vivo.

In this work, we evaluated the effects of intracortical microstimulation from electrode arrays coated with PEDOT/fCNT (referred to as PC from hereon) and IrOx using mesoscale fluorescence imaging and two‐photon imaging in awake, head‐fixed Thy1‐GCaMP6s mice. In particular, we aimed to examine and characterize differences in stimulation efficiency from these two materials determined by the cortical response amplitude, radius, and selectivity of the stimulated region to various stimulation paradigms. In addition, to facilitate our understanding of stimulation efficiency, we simulated the electric field generated by applied currents through geometric models that mimic the topography of PC and IrOx.

## 2 Results

### 2.1 Electrode Modification and Characterization

**Figure 1:**
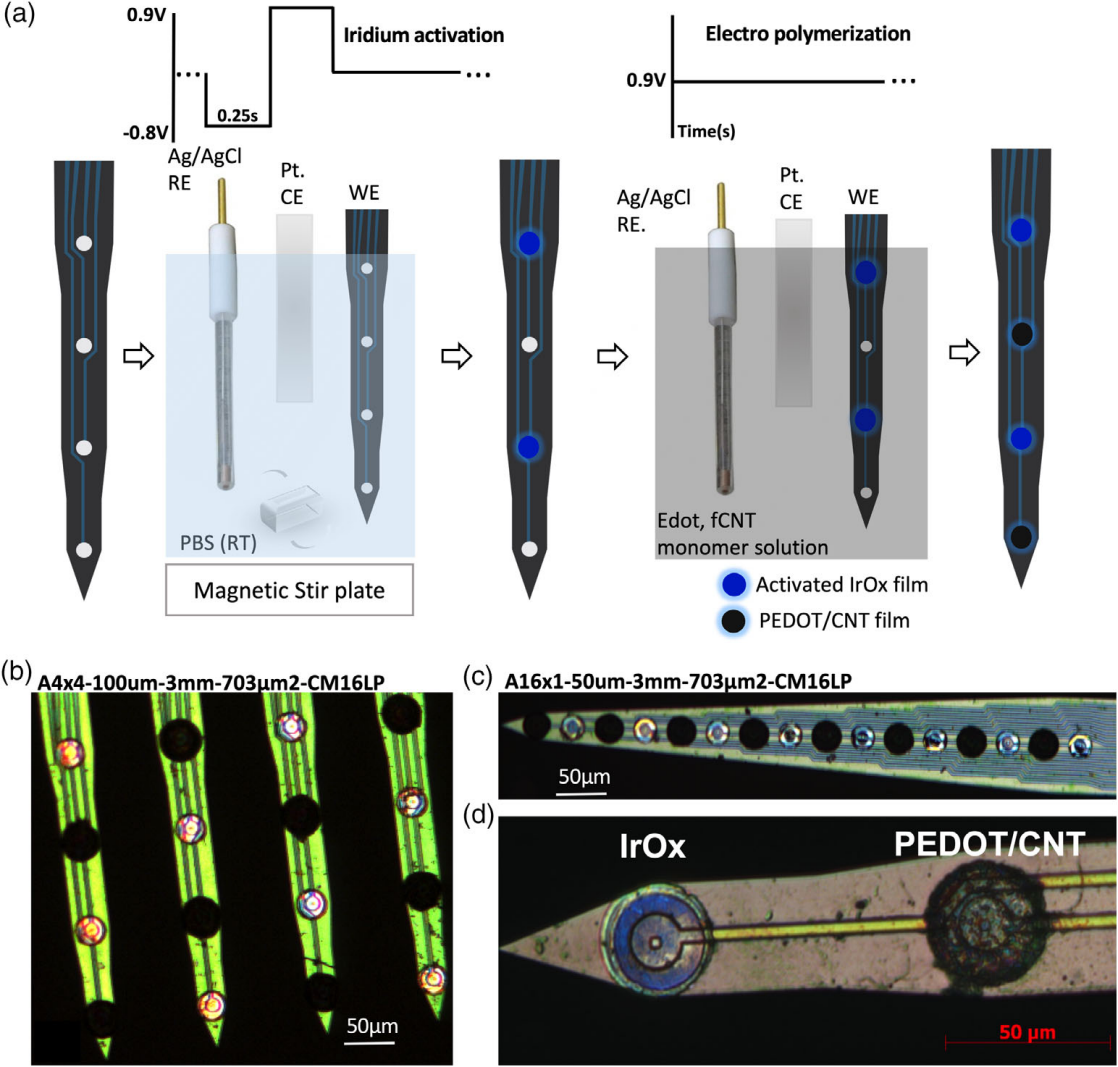
Electrode modification. a) Schematic depicting the electrode modification procedure. b,c) Optical micrograph of four‐shank and single‐shank electrodes with alternating sites coated with IrOx or PC. d) Higher magnification of IrOx and PC sites showing surface features under a bright‐field microscope. IrOx appears blue and PC appears black and fuzzy.

Electrode modification consisted of first electrically activating the iridium to form IrOx thin films on half of the sites, followed by coating on alternating sites with PC (**Figure** **1**a, see Experimental Section for details). IrOx and PC were coated on four‐shank (Figure 1b) and single‐shank(Figure 1c) arrays, respectively. Modified electrode sites have contrasting features under a bright‐field microscope, IrOx thin films appear blue and PC coatings have a characteristic black and fuzzy appearance (Figure 1b–d).

**Figure 2:**
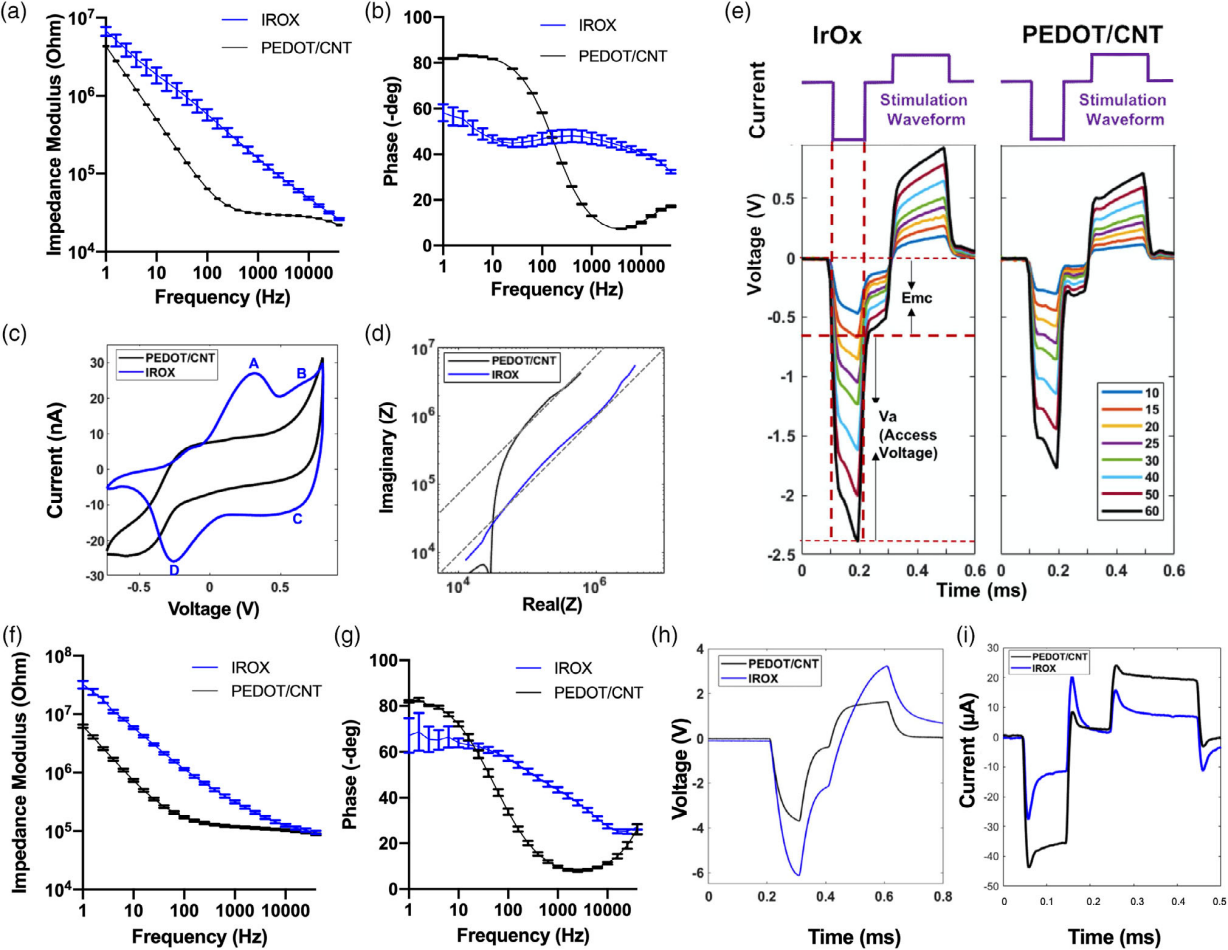
Electrochemical characterization. a,b) Impedance modulus and phase of IrOx and PC (denoted PEDOT/CNT in the legends) electrodes (*n* = 8 for each material type) in PBS. Error bars represent the standard error of the mean. c) Cyclic voltammetry of a representative PC and IrOx electrode site in PBS. A, D, B, C denote the oxidation and reduction peaks of iridium between Ir^3+^ to Ir^4+^ and Ir^4+^ to Ir^5+^ states, respectively d) Mean Nyquist plot of PC and IrOx sites (*n* = 8 for each material type) in PBS. e) Voltage excursions of a representative IrOx site (left) and a PC site (right) in response to current pulses from 10 to 60 μA in PBS (colored legends). Va represents access voltage and Emc represents the electrode polarization voltage used for the calculation of CILs. f,g) Impedance modulus and phase in vivo. h) In vivo voltage transient as a result of a 30 μA stimulus. All black traces represent PC and all blue traces represent IrOx except for panel (e). i) Current excursion as a result of a 3 V biphasic pulse measured at a representative PC and IrOx site. Error bars represent standard error of the mean.

Before implantation, the electrochemical properties of the IrOx and PC were characterized since modified electrodes have distinct electrochemical signatures (**Figure** **2**).

Figure 2a,b shows the impedance modulus and phase of IrOx and PC‐coated sites, respectively. The impedance modulus of IrOx sites was reduced with increases in frequency and the absence of a frequency‐independent region. In addition, the IrOx sites exhibited a relatively stable phase shift ranging between 60° and 30° over the frequency spectrum. In contrast, PC sites showed significantly lower impedance modulus than IrOx, with a frequency‐independent region of impedance modulus between 100 and 40 kHz, as well as a shift in phase angle from 80° at low frequencies to ≈15°–20° at high frequencies. The lower cut‐off frequency of PC (the frequency at which the impedance becomes purely resistive) indicates a higher capacitive charge transfer likely due to the increased effective surface area of the PC electrodes which is consistent with the scanning electron microscopy (SEM) observation (Figure S1, Supporting Information). Figure 2d shows Nyquist plots of PC and IrOx. The linear region for both materials represents a constant phase element with a phase angle of 45°, indicating a highly contoured surface. Moreover, we characterized the IrOx and PC coating with cyclic voltammetry (CV). Figure 2c shows a representative CV curve of an IrOx site (blue) and a PC site (black). PC CV curves demonstrate a shape that is stereotypical of PEDOT‐based coatings in that the curve has broad shoulders and no distinct redox peaks within the CV potential limits (−0.7 to 0.8 V). In contrast, IrOx undergoes reversible oxidation and reduction within this potential window. Particularly, peaks A, C indicate the oxidation (0.3 V) and reduction (−0.25 V) of iridium between Ir^3+^ and Ir^4+^ states, respectively; peaks B, D indicate the oxidation and reduction of iridium at 0.6 V between Ir^4+^ and Ir^5+^ states, respectively. The charge storage capacity is similar between the two site materials. Relevant electrochemical quantifications are shown in **Table** **1**. Voltage transients to applied current are shown in Figure 2e. Here, we applied biphasic waveforms in which the cathodic phase and anodic phase deliver an equal amount of charges. Charge balancing has been considered beneficial for safety as it eliminates net charge injection, which could cause tissue damage.^[^ ^63^ ^]^ As symmetric charge‐balanced waveforms may result in high anodic potentials that cause electrode corrosion,^[^ ^63^ ^]^ we can avoid this issue by having a higher amplitude in the cathodic phase and half the amplitude and double the duration in the anodic phase such that the anodic phase results in voltage transients lower than the oxidation window that may result in electrode corrosion.^[^ ^24^ ^]^ This asymmetric pulse paradigm has been applied in many animal and human microstimulation applications,^[^ ^12^, ^64^, ^65^ ^]^ thus was chosen for all of our stimulation waveforms. CIL for IrOx were estimated by dividing the cathodic charge at which *E*_mc_ reaches −0.6 V by the geometric surface area of the electrode (Table 1). The protruding underlying iridium contact makes it challenging to confine the electrodeposition to be within the electrode edge resulting in a slightly larger PC surface (≈1.25 times larger than IrOx sites). For this reason, CSCc and CIL values were also normalized to the new geometric surface areas of PCs and denoted with asterisks. In vivo measurements of IrOx and PC electrodes show similar electrochemical features as observed in vitro (Figure 2f,g). In addition, the peak‐to‐peak voltage in response to current pulsing results in a smaller amplitude than IrOx. Furthermore, by pulsing both materials with the same biphasic voltage pulse, the resulting current excursion shows a higher peak‐to‐peak amplitude in PC than IrOx (Figure 2i).

**Table 1:** In vitro electrochemical properties. The “*” denote values normalized to the enlarged PC surfaces

	Charge Storage Capacity [mC cm^−2^]	Impedance at 1 kHz [kΩ]	Charge Injection Limit [mC cm^−2^]
IrOx	25.1 ± 1.9	136 ± 9.3	1 ± 0.12
PC	26.4 ± 0.4; 21.12 ± 0.3*	45 ± 0.13	5.68 ± 0.13; 4.54 ± 0.1*

### 2.2 Mesoscale Imaging of GCaMP Response to Electrical Stimulation

**Figure 3:**
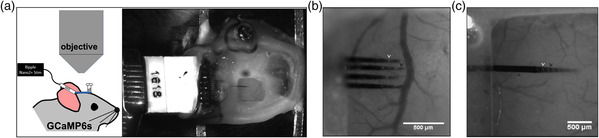
In vivo experimental setup. a, left) Cartoon of a GCaMP 6s mouse implanted with a multielectrode array at a 30° angle, the cranial window is sealed with a cover glass allowing for imaging of cortical responses. a, right) Optical micrograph of the cranial window. The electrode is affixed to a rectangular metal bar which was cemented with a contralaterally implanted stainless‐steel reference screw. b,c) Zoomed‐in view of the cranial window showing a four‐shank and a one‐shank electrodes array, respectively. White arrows point to IrOx sites and black arrows point to PC sites. Scale bars represent 500 μm.

We coated the conducting sites of NeuroNexus multielectrode arrays with IrOx and PC and implanted them into the somatosensory cortex of GCaMP6s mice. The arrays were interfaced with the stimulation hardware via a 16‐channel Omnetics connection. We stimulated each electrode site with biphasic, charge‐balanced, cathodic leading pulses ranging from 0.5 to 6 nC ph^−1^. Stimulations were administered at random for low (0.5–4 nC ph^−1^) and high (4.5–6 nC ph^−1^) charge densities for 1 s on and 3 s off while the animal is awake and head‐fixed on a treadmill (**Figure** **3**). Evoked neuronal activity for both probe styles was measured via GCaMP fluorescence changes through the cranial window (Figure 3b,c).

**Figure 4:**
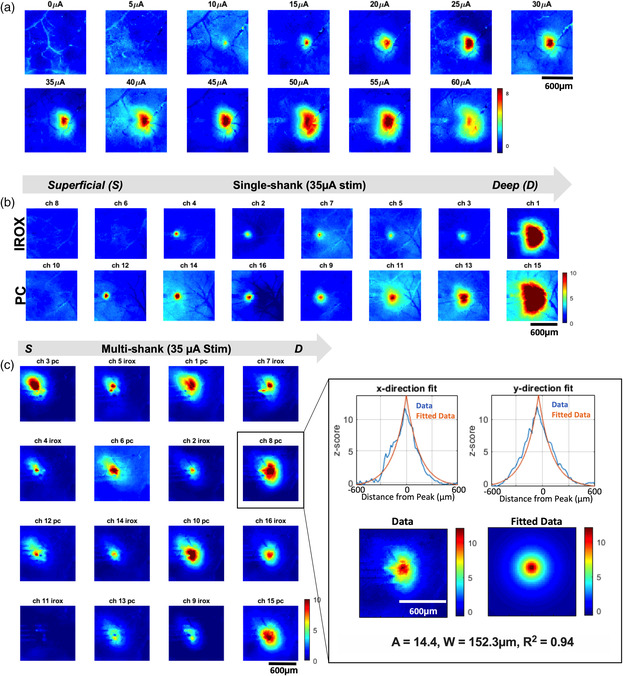
Mesoscale imaging of GCaMP response to electrical stimulation. a) Snapshots of the mean of six maximum GCaMP response to increasing electrical stimulation from a representative PC‐coated electrode site. The stimulation waveform was a biphasic, charge‐balanced, cathodic leading pulse (cathodic pulse width: 100 μs, interphase interval:100 μs, anodic phase: 200 μs, applied at 50 Hz) b) Snapshots of mean of six maximum GCaMP responses to a 35 μA stimulation on electrode sites from a single‐shank array. The top row shows responses from IrOx sites. The bottom row shows responses from their following (distal from the connector) PC sites, 50 μm away. c) Snapshots of the mean of six maximum GCaMP responses to a 35 μA stimulation on electrode sites from a four‐shank array whose alternating sites are coated with PC or IROX. Inset shows a representative 2D exponential fitting from the maximum GCaMP response of an individual trial from Ch8. The fit was carried out on the individual trial data, profiles of the fit are shown on the top row along the *x*‐ and *y*‐directions (blue is the experimental data and orange is the model fit). The bottom row shows the 2D experimental image data (left) and model fit in the 2D image (right). The result of the fitting is represented in the exponential decay equation with the peak GCaMP response of 14.4 (*z*‐score) and radius of activation of 152.3 μm. Gray arrows denote electrode positions from superficial to deep. Scale bars represent 600 μm.

The GCaMP fluorescence increased with increasing electrical stimulation intensity (**Figure** **4**a). In addition, stimulating via sites of different coating materials within the same electrode array resulted in heterogeneous GCaMP responses (Figure 4b,c). We quantified the intensity and radius of GCaMP responses using a 2D exponential curve (Figure 4c inset). The height and width of the exponential curve represent the intensity and radius of the GCaMP response.

**Figure 5:**
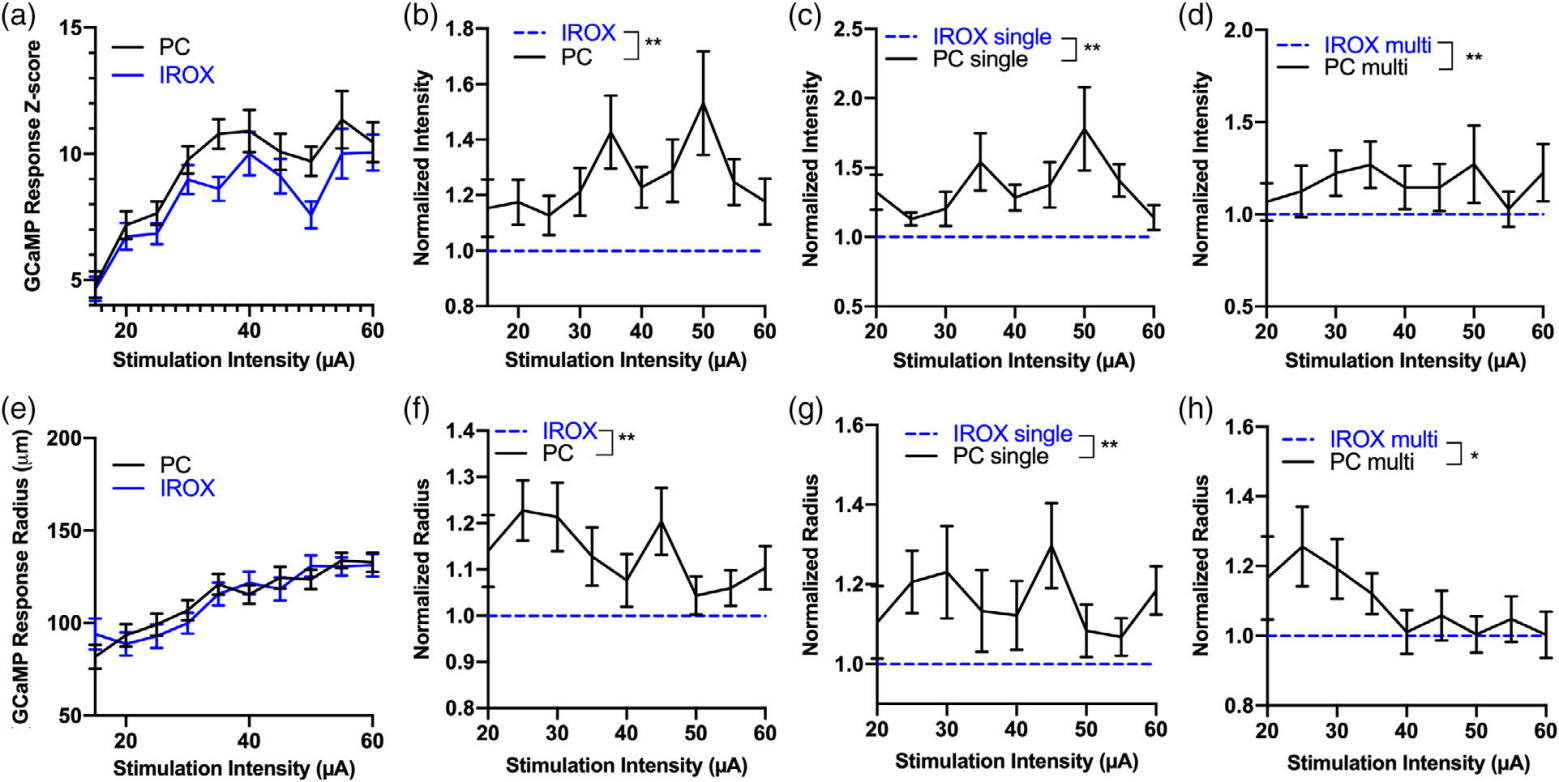
Quantification of GCaMP response to electrical stimulation. a) Mean GCaMP intensity as a function of stimulation amplitude (pooling data from single and multishank arrays). GCaMP intensity increases with increasing stimulation current. PC sites elicit significantly higher GCaMP response than IrOx sites. **p < 0.005, two‐way ANOVA. *n* = 44–60 electrode sites that elicited threshold crossing GCaMP intensity at different stimulation amplitudes. *N* = 10 mice. b) Mean normalized GCaMP intensity as a function of stimulation amplitude regardless of electrode geometry. **p < 0.005, Wilcoxon signed‐rank test. c) Mean normalized GCaMP intensity as a function of stimulation amplitude for both materials for single‐shank electrode arrays. *n* = 20–30 electrode sites that elicited threshold crossing GCaMP intensity at different stimulation amplitudes. *N* = 5 mice. ** *p < 0.005,* Wilcoxon signed‐rank test. d) Mean normalized GCaMP intensity as a function of stimulation amplitude for both materials for multishank electrode arrays. *n* = 14–26 electrode sites that elicited threshold crossing GCaMP intensities at different stimulation amplitudes. *N* = 5 mice. **p < 0.005, Wilcoxon signed‐rank test. e) The mean GCaMP radius increases as a function of stimulation amplitude for both material types. *n* = 44–60 electrode sites that elicited threshold crossing GCaMP response at different stimulation amplitudes. *N* = 10 mice. f) Normalized radius from (e). PC sites elicited significantly broader GCaMP responses compared with IrOx sites. g) The normalized radius for single and h) multishank electrode arrays. * *p < 0.05,* Wilcoxon signed‐rank test. All error bars represent the standard error of the mean.

Quantification of the mesoscale imaging confirms the observation that increases in stimulation intensity resulted in increasing neuronal activation as represented by the GCaMP response intensity and radius (**Figure** **5**a,e). The increase in GCaMP response amplitude suggests an increase in the density of neurons in the vicinity of the electrode, meanwhile, the increase in GCaMP radius suggests an increase in activation volume. We then examined the potential differences in material. Due to the heterogeneous expression of GCaMP and neuronal distribution across the cortical depth, we normalized GCaMP intensity and radius elicited by PC sites to their IrOx counterpart. PC sites stimulated significantly more intense GCaMP activity across the amplitudes that were applied (*p < 0.005*). In addition, this observation was consistent across the two electrode geometries (Figure 5c,d). Furthermore, PC sites elicited a significantly higher GCaMP radius, regardless of probe geometry (Figure 5f–h).

### 2.3 Effect of the Number of Electrode Shanks on GCaMP Intensity and Radius

**Figure 6:**
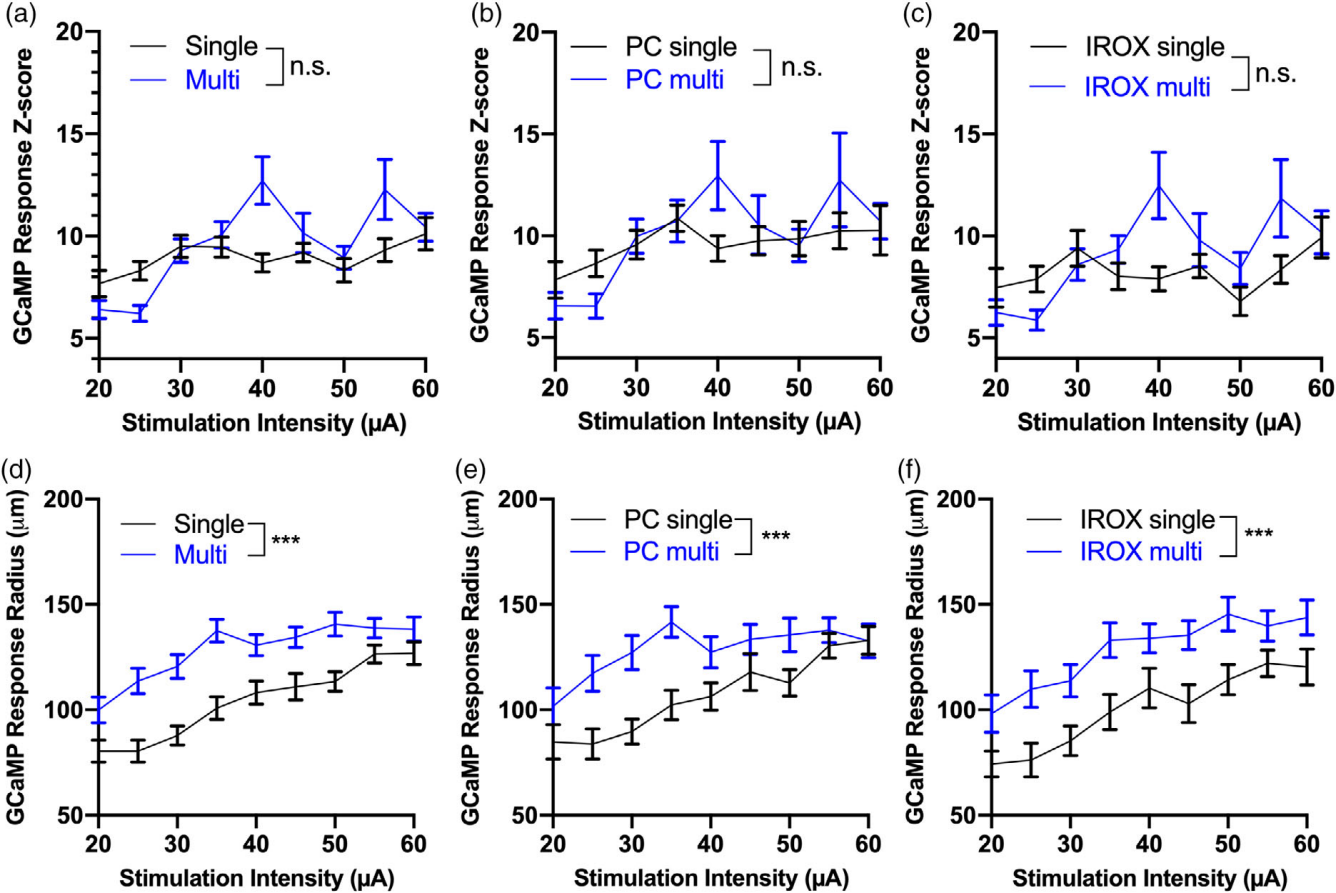
Effect of electrode geometry on GCaMP intensity and radius. a) Mean GCaMP intensity as a function of stimulation amplitude for single and multishank electrode arrays. Pooling data from both materials. *n* = 40–59 electrode sites that elicited threshold‐crossing GCaMP events in 5 mice, for each geometry type. b) Mean GCaMP intensity as a function of stimulation amplitude from PC sites in single versus multishank arrays. *n* = 19–30 electrode sites that elicited threshold crossing GCaMP events in 5 mice, for each geometry type. c) Mean GCaMP intensity as a function of stimulation amplitude from IrOx sites in single versus multishank arrays. *n* = 21–30 electrode sites that elicited threshold crossing GCaMP events in 5 mice, for each geometry type. d) Mean GCaMP radius as a function of stimulation amplitude, pooling data from both materials. Multishank arrays stimulate a higher radius of activation compared to single‐shank arrays. ***p = 0.0005, two‐way ANOVA, *n* = 40–60 electrode sites that elicited threshold crossing GCaMP events in 5 mice, for each geometry type. e) Mean radius of activation as a function of stimulation intensity for single versus multishank arrays elicited by PC sites. ***p = 0.0001, two‐way ANOVA. *n* = 19–29 electrode sites that elicited threshold‐crossing GCaMP events in 5 mice, for each geometry type. f) Mean radius of activation as a function of stimulation intensity for single versus multishank arrays elicited by IrOx sites. ***p = 0.0005, 20–30 electrode sites that elicited threshold crossing GCaMP events in 5 mice, for each geometry type. All error bars represent the standard error of the mean.

The number of probe shanks did not significantly alter GCaMP intensity with increasing stimulation amplitude (**Figure** **6**a). This observation is consistent across material types (Figure 6b,c). In contrast, stimulating from multishank arrays resulted in significantly broader GCaMP activity than stimulating from single‐shank arrays (Figure 6d). This observation is consistent across both material types (Figure 6e,f).

### 2.4 Material Differences in Constant‐Voltage Versus Constant‐Current Stimulation

**Figure 7:**
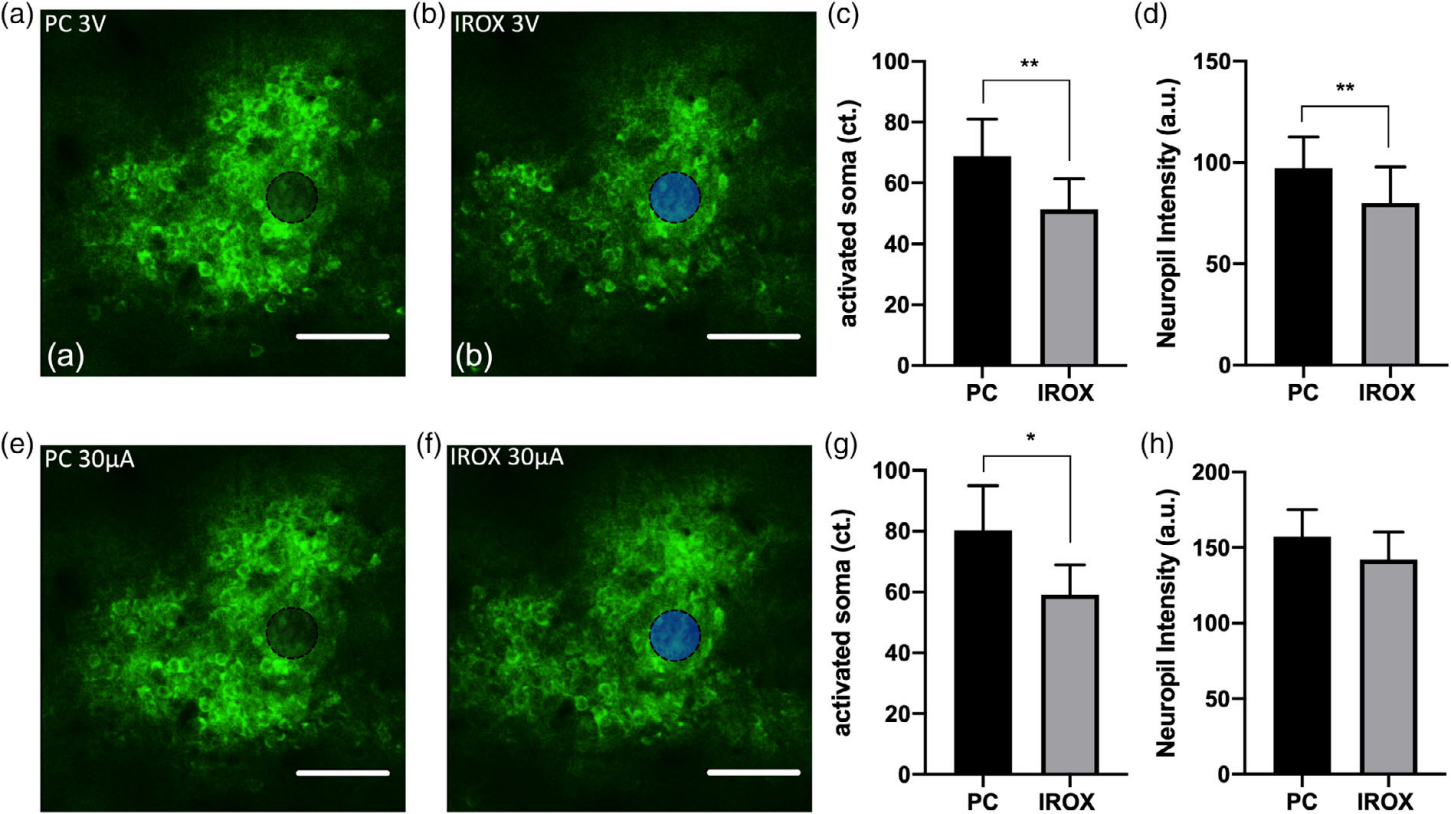
TPM investigation of voltage‐ and current‐controlled electrical stimulation. a,b) Neural elements evoked by 3 V biphasic voltage‐controlled stimuli from a PC and an IrOx, respectively. Electrode locations are denoted by black and blue disks, respectively. Scale bars represent 50 μm. c) Quantification of electrically activated neuronal soma as a result of a 3 V stimulus. **p < 0.01. d) Quantification of neuropil intensity within the image after subtracting somas. **p < 0.01. e,f) Neural elements activated by 30 μA biphasic current‐controlled stimuli from a PC and an IrOx. Electrode locations are denoted by black and blue disks, respectively. (g) Quantification of electrically activated somas from PC and IrOx sites. *p < 0.05. h) Quantification of neuropil intensity after subtracting somas. *n* = 10 sites for each material tested in *N* = 3 mice. All error bars represent the standard error of the mean.

We then examined whether electrode material plays a role in neuronal activation using either constant‐voltage or constant‐current stimulations. First, we delivered charge‐balanced biphasic voltage‐controlled stimulation at 3 V in the cathodic phase, which generated a maximum cathodic current of ≈28 and 43 μA for IrOx and PC electrodes, respectively (Figure 2i). The elicited calcium response was measured by TPM (**Figure** **7**a,b). Electrical stimulation via PC sites with a peak voltage of 3 V activated a significantly larger number of neuronal soma (*p* < 0.01) as well as significantly higher intensity of neuropil (*p* < 0.01) compared with IrOx (Figure 7c,d). Next, we examined the delivered charge‐balanced biphasic current‐controlled stimulation at 30 μA in the cathodic phase and imaged the GCaMP response (Figure 7e,f). Stimulation via PC sites activated a significantly higher number of somas (*p* < 0.05) compared with IrOx, whereas no significant difference in neuropil recruitment was observed (Figure 7g,h).

To compare the efficiency of electrical stimulation for IrOx and PC, we calculated the electrical energy for current‐controlled stimulation. PC electrode sites delivered significantly less energy compared with IrOx sites, (15 ± 0.4 nJ, *n* = 41 PC sites. 17.5 ± 0.5 nJ, *n* = 38 IrOx sites, *p* = 0.0005, unpaired two‐tailed *t‐test*). If we define stimulation efficiency as the number of neurons activated per Joule, the stimulation efficiency of PC is 80 ± 47 cells nJ^−1^, which is significantly higher than IrOx 60 ± 31 cells nJ^−1^ (*p < 0.05)*.

### 2.5 Pulse Width Modulation for Microstimulation Selectivity

**Figure 8:**
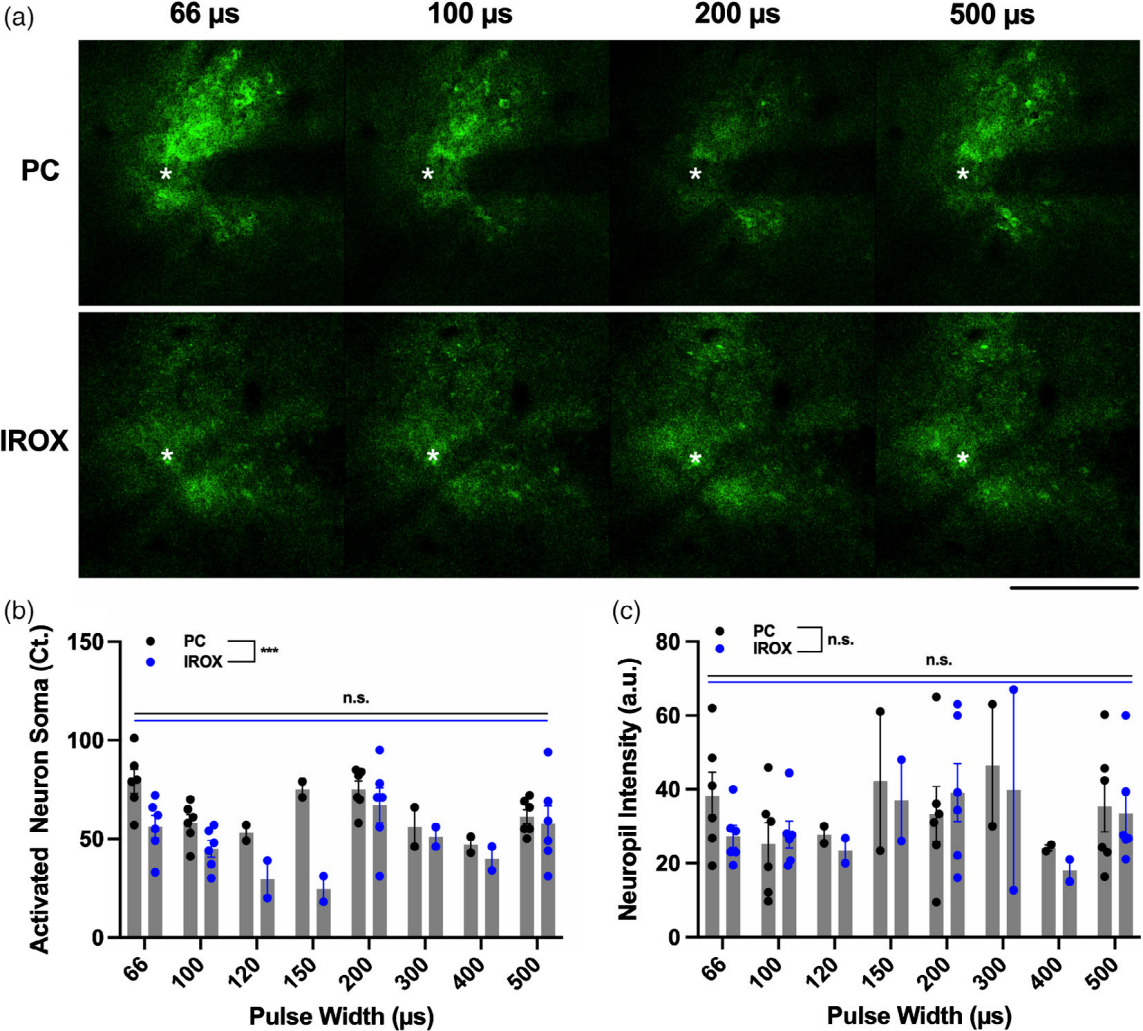
Effect of pulse width modulation on neural activation. a) Representative TPM images showing electrically stimulation GCaMP expressed neural elements for 66–500 μs pulse widths at 4nC ph^−1^ for PC and IrOx coatings. b) Quantification of electrically activated neuron soma for pulse widths between 66 and 500 μs for PC and IrOx coatings. PC activates significantly higher neuronal soma than IrOx across all pulse widths. There is no significant difference among the activated neuron soma among pulse widths for either material. Black and blue dots represent *n* = 2–6 biological replicates for each material for each pulse width pooled from N = 3 mice. Two‐way ANOVA. *** *p* = 0.0002. c) Quantification of electrically activated neuropil as a function of pulse widths for PC and IrOx coatings. There are no significant differences in neuropil activation between material types and pulse widths. Two‐way ANOVA. All error bars represent the standard error of the mean.

**Figure 9:**
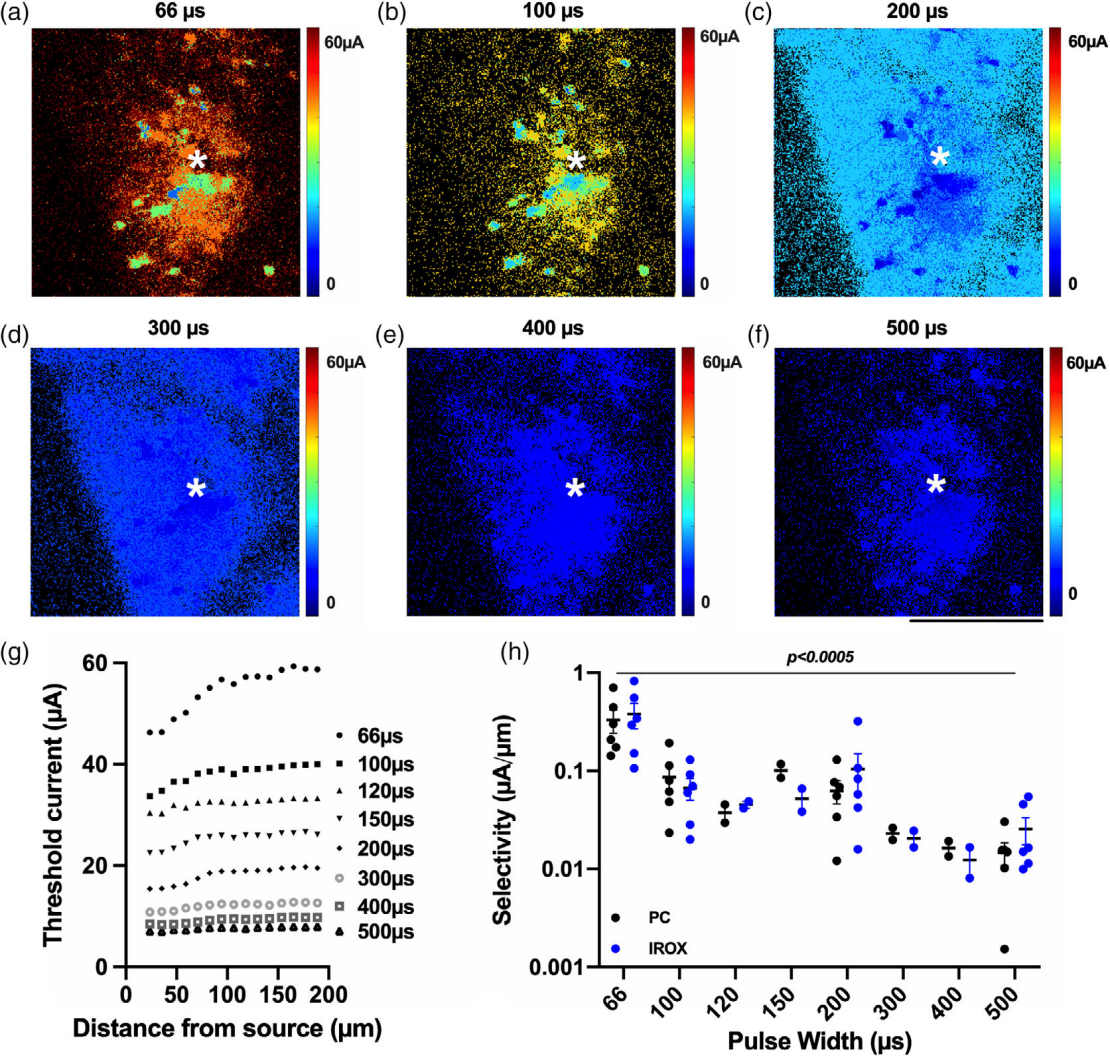
Effect of pulse width modulation on neuronal selectivity. a–f) Representative threshold maps for charge injections between 1 and 4nC ph^−1^ by varying pulse width and current amplitude. Electrode positions are denoted with asterisks. Color bars represent threshold currents for each pixel. g) Representative plot of threshold current as a function of distance away from the center of the electrode. Different marker styles represent different pulse widths. h) Selectivity significantly decreases with increasing pulse widths. Black dots represent selectivity values for each pulse width from *n* = 4–12 electrode sites *N* = 3 mice. *P*‐value represents the significance of the slope of the linear regression for selectivity versus pulse width. Scale bars represent 100 μm. All error bars represent the standard error of the mean.

Using TPM, we examined the effect of modulating pulse width and current amplitude while maintaining a constant charge between 1 and 4 nC ph^−1^, on neuronal recruitment. **Figure** **8**a shows representative TPM images of neural elements stimulated by 66–500 μs long cathodic pulses at 4 nC ph^−1^ using electrodes coated with PC and IrOx. PC‐coated electrodes activated a significantly higher number of neuronal soma than IrOx‐coated electrodes across all pulse widths. In addition, there were no significant differences in the number of activated neuron soma among pulse widths for either material (Figure 8b). Figure 8c shows the quantification of electrically activated neuropil as a function of pulse widths for PC and IrOx coatings. There were no significant differences in neuropil activation between material types and among pulse widths. **Figure** **9**a–f shows threshold maps of the same brain region stimulated by the same electrode (white asterisk) for different pulse widths. The color for each pixel represents the lowest current for it to be significantly activated by the electrical stimulation. We observed that stimulating with shorter pulses results in more spatially distinct neuronal recruitment. As the pulse width increases, the recruited neuronal elements become less distinguishable. We quantified this trend by binning each image from the center of the electrode to the edges of the ROI and calculating the average threshold current for each bin (Figure 9g). To compare the differences in selectivity among these pulse widths, we carried out linear regression for all recruitment curves within 100 μm from the electrode center where the relationship between threshold current and distance was the most linear, and all regressions exhibited significant nonzero slopes (data not shown). Moreover, by carrying out linear regression between selectivity and pulse width we observed that the increase in pulse width resulted in significantly decreased spatial selectivity (Figure 9h). The material type did not have an effect on pulse width modulated neuronal selectivity.

### 2.6 Finite Element Analysis of Electric Field

**Figure 10:**
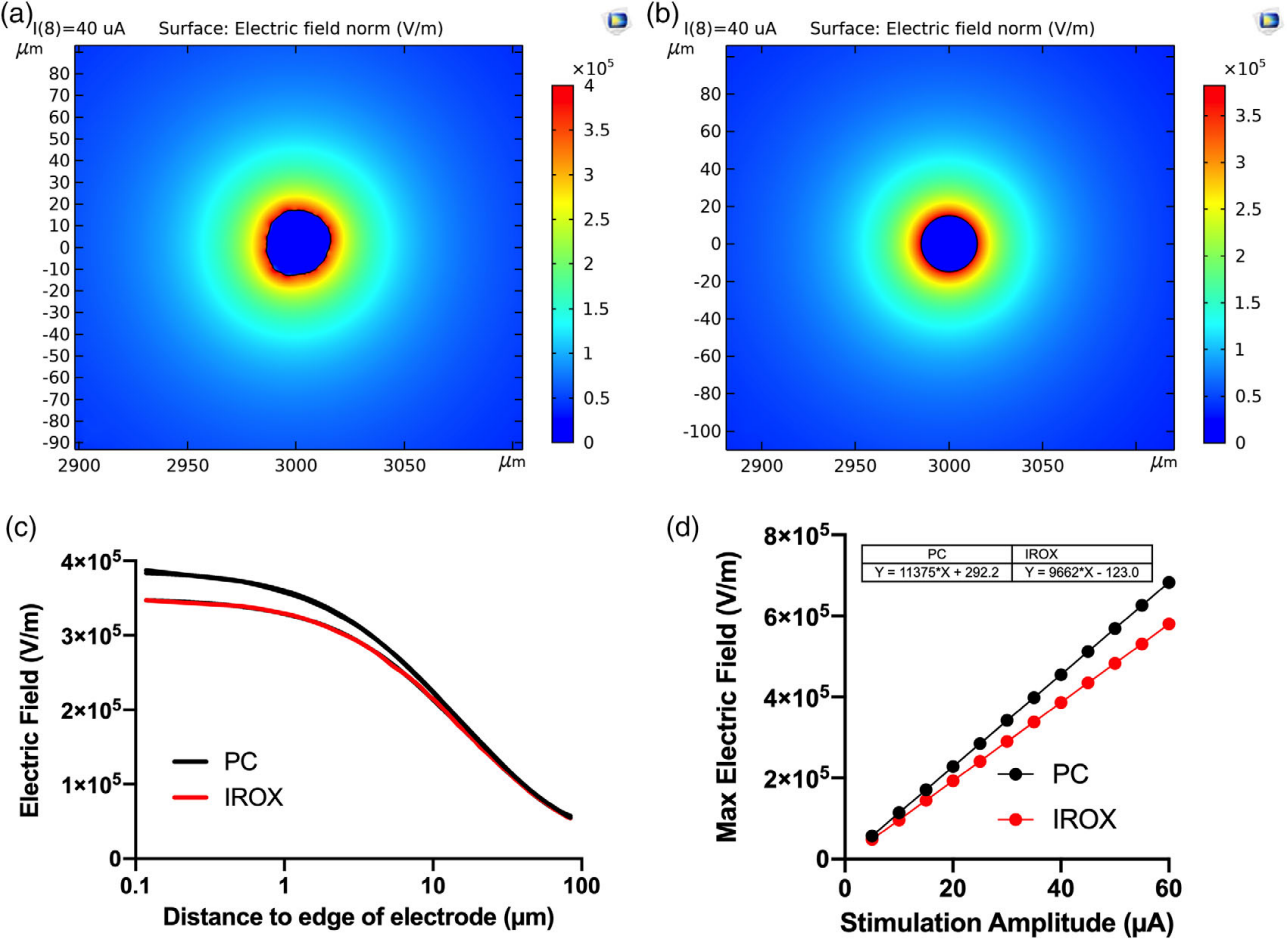
Finite element analysis of PC and IrOx electrode surfaces. a) 2D Visualization of electric field distribution of a PC surface with a spatial resolution of 76 μm^−1^ and spectral exponent of 1.5, as a result of a 40 μA stimulus referencing a far‐away stainless steel screw. b) 2D Visualization of electric field distribution of an IrOx surface simulated from a perfect disk, as a result of a 40 μA stimulus for a far‐away stainless steel screw. Both images are zoomed in near the electrode site. Color bars represent the electric field for (a,b). c) Electric field gradient as a function of distance away from the edge of the electrode. d) The maximum electric field strength along the surface of the electrode as a function of virtual stimuli applied between 5 and 60 μA.

Using COMSOL Multiphysics, we created a 2D model for PC and IrOx (**Figure** **10**) to help elucidate the potential mechanism for increased neural activation from PC‐coated electrodes. We hypothesize that the nanofibrous topography, or the roughness, of the PC surface, creates a stronger nonuniform electric field that increases the likelihood of additional activating neural elements compared with IrOx‐coated electrodes. Representative images of the surface electrical field for PC and IrOx at 40 μA current amplitude show that the rough edge of the PC has a more concentrated electric field adjacent to the electrode (Figure 10a) compared with the electric field immediately next to the edge of an IrOx electrode (Figure 10b). Examination of the voltage field profile as a function of distance from the edge of the electrode reveals that there are two phases to the change to voltage field next to the edge of the electrode, a slow‐ and a fast‐exponential decay. Table S1, Supporting Information, shows fitting parameters from the two‐phase exponential decay function. PC has an 11.4% larger absolute maximum electric field strength than IrOx. In addition, the nonuniform electric field strength nearby the rough PC surface caused a 21% higher amplitude of the slow decay phase (*A*_slow_) higher than the slow phase of the IrOx.

Furthermore, we applied increasing currents from 5 to 60 μA at both electrode sites and observed increasing maximum normal electric field for both materials, with PC having a higher slope than IrOx electrodes, i.e., 1.14 × 10^4^ and 0.97 × 10^4^, respectively.

## 3 Discussion

Recent advances in the use of microstimulation for restoring movement, sensation, vision, and hearing motivate the need for a deeper understanding of how electrode materials, stimulation modality, and stimulation parameters affect the efficiency, selectivity, safety, and stability of stimulation. In vivo imaging in awake GCaMP6s mice affords us the opportunity for direct visualization of the stimulated outcome in real time. Particularly, this study examined the efficiency and selectivity of PC‐coated electrodes for electrical microstimulation using in vivo imaging in comparison with the commonly used microstimulation material IrOx. We modified the individual microelectrode sites of NeuroNexus probes with IrOx and PC coatings in an alternating pattern to directly compare the two electrode materials. First, we delivered electrical stimuli ranging from 0.5 to 6 nC ph^−1^ and used a model to obtain a measure of the GCaMP response amplitude and radius from mesoscale fluorescence microscopy data. PC coated electrodes evoked significantly stronger GCaMP response amplitudes and larger radii compared with IrOx. Next, using TPM, we examined whether electrode material plays a role in the magnitude of neuronal activation and energy delivery in constant‐voltage and constant‐current stimulations. PC‐coated electrodes exhibited superior energy efficiency for neural stimulation in terms of activating more intense and broader GCaMP for the same delivered charge compared with IrOx‐coated electrodes. Meanwhile, also using TPM, we explored the effect of pulse width modulation on cortical stimulation selectivity using IrOx and PC. Stimulation with shorter cathodic leading pulse widths resulted in more selective neural activation compared with longer pulses for both materials. Finally, using finite element analysis, we investigated the differences in electric field imparted by the two materials. The nanofibrous topography of PC resulted in a higher electric field strength than IrOx, increasing the likelihood of activating nearby neural elements.

### 3.1 Electrochemical Features of PC and IrOx

Characterization of the modified electrodes reveals unique electrochemical signatures of the two electrode materials. PC‐coated electrodes had significantly lower impedance, higher CIL and similar cathodic charge storage capacity compared with IrOx‐coated electrodes. Consistent with observations in the literature, the unique electrochemical properties of PC are attributed to the nanofibrous structure of the coating, dramatically increasing the electrochemical surface area for charge transfer,^[^ ^33^, ^44^ ^]^ compared with the IrOx surfaces, which are relatively smoother. Other methods for forming IrOx such as sputtered IrOx films can result in a nanosurface topography which increases electrochemical surface areas and improves the electrochemical behavior.^[^ ^21^, ^23^ ^]^ Also, to be consistent with most of the stimulation paradigms used in animal and human studies, we did not perform a bias voltage on IrOx coated electrodes during stimulation, which has been shown to further reduce impedance and improve the CIL.^[^ ^24^ ^]^ In vivo, both electrode materials retained their electrochemical features as seen in vitro (Figure 2f,g). The overall elevated impedance is due to the higher impedance of tissue compared with phosphate buffered saline (PBS). In vivo current pulsing at 30 μA to both materials resulted in a voltage transient with lower overall amplitude for PC electrodes (Figure 2h). Lower in vivo voltage transient and lower *E*_mc_ suggest a higher charge injection capacity before the safety limit is reached for the PC electrode. Furthermore, less energy is consumed from the PC electrode than IrOx at the same current injection, which is desired for extending battery life in chronic constant‐current stimulators with implanted batteries. Similarly, due to the lower electrical impedance offered by PC surfaces, pulsing both materials with a 3 V biphasic stimulus results in a higher injected current, offering potential higher power efficiency compared with IrOx surfaces.

### 3.2 Effect of Electrode Material on the Intensity and the Spread of GCaMP Response

Fluorescence microscopy in awake head‐fixed mice provides direct visualization of cortical responses to electrical stimulation. Lower stimulation amplitudes (0.5–1.5 nC ph^−1^) showed nonstatistically significant GCaMP responses that were indistinguishable from ongoing GCaMP fluorescence fluctuations in awake mice. Increasing charge density between 2 and 4 nC ph^−1^ resulted in increasing GCaMP responses (Figure 5a). This is expected because increasing charge injection increases the extracellular voltage which increases the likelihood of initiation of the action potential of nearby neural elements.^[^ ^66^ ^]^ In addition, the rate of increase in GCaMP intensity stimulated by the PC electrode is higher than that stimulated by the IrOx electrode. This phenomenon could be partially explained by our modeling result in that the rough electrode surface creates a higher voltage field immediately next to the electrode than the IrOx surface. However, GCaMP responses did not continue to increase for charge densities between 4.5 and 6 nC ph^−1^ (Figure 5a), for both electrode materials. As there was no significant increase in electrical impedance for both materials before and after stimulation, we exclude the unlikely contribution of changes in electrode property during stimulation (Figure S4, Supporting Information). This phenomenon may be attributed to acute neuronal injury due to electrode implantation. The insertion of the electrode into the cortex triggers acute inflammation which deprives the extracellular environments of nutrients such as oxygen and glucose, which are essential for action potential initiation,^[^ ^67^ ^]^ as such the neuronal elements are not able to meet the high metabolic demand of high‐intensity stimulation. In addition, the severed axonal connections and membranes of somas likely contributing to the plateau in recruiting neural elements.

Due to the heterogeneity of GCaMP6s expression and neuronal density relative to cortical depth (Figure 4b,c), the comparison of electrode efficiency was made by normalizing the PC site data to their neighboring IrOx counterparts, resulting in up to eight pairs of comparisons per mouse. Statistical analyses revealed that PC sites elicited significantly higher and broader GCaMP responses than IrOx sites for both electrode shank densities (Figure 5). Due to the nanofibrous nature of the PC coating, microscale surface topography creates a significantly rougher surface compared with IrOx coatings. The increase in surface roughness results in 1) increased nonuniform voltage distribution, thereby increasing the activating function of nearby neural elements,^[^ ^68^ ^]^ and 2) the 3D morphology of the PC coating reducing the distance between the current source and neural elements. Using COMSOL, we created a 2D finite element model of a rough surface mimicking the fractal features of PC and compared the electric field distribution profile as a result of an applied current to that of a smooth disk electrode. Similar to findings in other modeling studies estimating electric field distribution of fractal electrode designs,^[^ ^68^, ^69^, ^70^ ^]^ there was a significantly stronger electric field immediately nearby the PC electrode.

### 3.3 Effect of Probe Shank Number on GCaMP Response

We examined the effect of probe shank number on cortical responses to electrical stimulation. There were no significant differences between the electrode shank number on GCaMP responses (Figure 6a) for either electrode material (Figure 6b,c). Although the radius of activation increased with increasing stimulation intensity (Figure 6d), the overall radius of activation elicited by multishank arrays was higher than that elicited by single‐shank arrays. (Figure 6e,f). This could be explained by the larger insertion footprint from multishank arrays relative to single‐shank arrays, resulting in larger areas of the brain with damaged neural elements. Our group has previously investigated calcium activity and morphology of neurons before, during, and 1 month after the insertion of an electrode array, and found that the implantation leads to sustained, abnormal high calcium levels in neurons within 150 μm of the implant. These neurons were morphologically distorted, and some cellular membranes were mechanoporated which could increase the likelihood of calcium influx. Neurites exhibited signs of axonal injury in response to the device implantation, forming swollen, hypertrophic spherical bodies, or “blebs.”^[^ ^58^ ^]^ Also, axotomy as a result of mild traumatic brain injury has been reported to increase neuronal network excitability within 48 h after damage,^[^ ^71^ ^]^ which could explain the more excitable brain environment around multishank probes compared with single‐shank probes acutely. While the healthy calcium activity recovers over the first month in this study, how the initial damage, especially by electrodes with larger footprints such as the Utah 10 × 10 multielectrode arrays, affects the long‐term neuronal function for electrical stimulation, remains to be investigated.

### 3.4 Higher Stimulation Efficiency from PC than IrOx in Both Voltage and Current‐Controlled Stimulation Modalities

There are two types of stimulation modalities for cortical microstimulation: constant‐voltage stimulations and constant‐current stimulations. In constant‐voltage stimulations, voltage is applied between the microelectrode and the ground resulting in an injected charge into the tissue. However, due to foreign body response, the conductivity of the local environment of the electrode can vary throughout the implant period. This will result in unpredictable charge injection, making it difficult to maintain stimulation consistency. On the other hand, constant‐current stimulations allow us to control the total injected charge, making chronic microstimulation more reliable with an increased risk of voltage transient exceeding the water window. For TPM studies, the GCaMP response within the field of view consists of excitatory neuron somas at the imaging plane, and neuropil, which consists of axons, dendrites, and out‐of‐plane somas. We observed a significant advantage in both neuronal soma and neuropil recruitment for PC coated electrodes compared with IrOx electrodes for constant‐voltage stimulation. This observation agrees with our understanding that a lower impedance electrode results in higher injected current amplitude (Figure 2i).^[^ ^45^ ^]^ In contrast, we have observed a significant difference in neuron soma recruitment but no significant difference in neuropil activation in the constant‐current mode from the two materials. The difference in soma activation likely stems from the increased activating function of neurons as a result of an increased and nonuniform voltage distribution nearby PC sites.^[^ ^68^, ^70^ ^]^ Also, the 3D nanofibrous network of the PC coating enables a more intimate connection with the neural tissue compared with the relatively smoother IrOx surface. Closer contact between the electrode and the excitable neural tissue, reduces the likelihood of current shunting by the extracellular fluid, resulting in more efficacious neural stimulation. The lack of statistical significance in the neuropil activation could be explained by the small difference in extracellular voltage elicited by both materials at 30 μA (Figure 10). In addition, with our two‐photon imaging set up, we can only observe neural elements within one plane for each stimulation session, where the majority of fluorescence are contributed by the highly GCaMP expressing neuronal soma, which could influence the detection of statistical differences in neuropil intensity upon stimulation from both electrode materials. Regardless, for current‐controlled stimulation, PC electrodes delivered significantly less energy compared with IrOx, making them a more energy‐efficient material for battery life preservation in chronically implanted stimulators.

### 3.5 Pulse Width Modulation on Stimulation Selectivity in the Cortex

In the realm of therapeutic and functional stimulations of the CNS, pulse width modulation has been associated with various functional outcomes in therapeutic efforts. In auditory prostheses, modulating pulse widths affected auditory percepts other than loudness, particularly, a longer pulse duration (266 μs) resulted in a different perceived pitch than a short pulse duration (50–100 μs).^[^ ^72^ ^]^ In retinal prostheses, focal cellular responses could be achieved with relative short pulse durations (≤120 μs), which improves the spatial resolution and more ideal shape perception.^[^ ^65^ ^]^ In studies in nonhuman primates for sensory prostheses, the detection threshold was lower for shorter pulse widths compared with longer pulse widths, however, this difference disappears at higher stimulation frequencies.^[^ ^73^ ^]^ In human cortical microstimulation, pulse widths between 50 and 400 μs have been surveyed in the motor and the sensory cortices. For somatosensory restoration studies in humans, 200 and 400 μs pulse widths elicited two different types of sensations, electrical buzz, and tingling, respectively.^[^ ^74^ ^]^ We investigated the effect of pulse width modulation on neuronal activation selectivity using TPM. We maintained a constant charge between 1 and 4 nC ph^−1^ and varied the pulse width and current amplitude. We quantified the activation profile of layer II/III neurons. The threshold current maps of neuronal responses at different pulse widths reveal more spatially distinct activations with shorter pulse widths compared with longer pulse widths (Figure 8). A potential explanation for this observation is that 1) for the same charge density (nC ph^−1^) there is a higher temporal current density (μA μs^−1^) delivered with shorter pulses compared with longer pulses, and 2) with increasing charge densities, there was a higher increase in current amplitude with shorter pulses compared with longer pulses. Both factors can increase the likelihood of activating fibers with varying diameters and fibers that are farther away from the current source, resulting in a more spatially distinct activation with shorter pulses. Our observations are consistent with reports made in the ex vivo calcium imaging study investigating stimulation strategies for selective activation of retinal ganglion cells.^[^ ^65^ ^]^ In their study, using charge‐balanced cathodic leading pulses, stimulating with pulse widths beyond 500 μs yields spatially indistinguishable activation of neural elements at increasing current amplitudes.

### 3.6 Limitation and Future Studies

While our study has the advantage of stimulating and imaging the cortex of awake mice which mimics the conditions of human studies, the awake brain had large ongoing fluctuations. These large fluctuations made it difficult to capture significant mesoscale GCaMP responses at low stimulation amplitudes (5–15 μA). However, we were able to accurately depict GCaMP activation patterns at amplitudes higher than 15 μA and drew comparisons between materials. In addition, simulating human studies, our stimulation paradigms did not involve the use of bias voltages which has been demonstrated beneficial for increasing the conductivity and CIL of IrOx electrodes.^[^ ^24^ ^]^ Moreover, while implantation at a 30° angle allows imaging of electrically stimulated neural response, this setup is still limited to superficial regions of the cortex. The depth of imaging is further obstructed by the radio‐opaque Michigan arrays. The use of three‐photon microscopy^[^ ^75^ ^]^ and the use of transparent arrays^[^ ^76^ ^]^ can increase image depth and image quality. Future research should explore the long‐term stability of these electrode materials and the long‐term stability of stimulation efficiency and investigate the functional and behavioral outcomes in several animal models. Furthermore, there are several limitations to the modeling study. The self‐affinity features of PC will depend highly on the scale they are investigated in. The derivation of topographical features in our study was from SEM images at the microscale whose spectral exponent resulted in a visually apparent rough topography in the COMSOL model. In addition, the 2D simulation of electric field strength may be a modest estimation of the true electric field strength of the 3D nanofibrous topography of PC, closer approximations of the 3D electrode topography may be achieved by carrying out AFM and nano‐computed tomography.^[^ ^77^ ^]^ Furthermore, our simulation assumes a perfect brain environment with homogenous conductivity, which does not describe the true in vivo environment with different zones of tissue compositions with varying conductance values surrounding the electrodes. Future work should incorporate these considerations along with modeling the probability of neural activation using NEURON for a more complete picture.

## 4 Conclusion

With the increasing development of efficient materials for electrical stimulation, it is crucial to understand their in vivo performance. We investigated the in vivo stimulation efficiency of two high charge injection materials. Using advanced imaging techniques, we observed distinct differences in neuronal responses stimulated by PC and IrOx coatings. Specifically, we observed significantly higher and broader neural activation by PC‐coated electrodes compared with IrOx‐coated electrodes. This observation was highly likely due to 1) the nanofibrous surface topography of PC electrodes, creating a more nonuniform electric field compared with IrOx electrodes, thereby increasing the activating function of nearby neural elements, and 2) the fractal nature of the PC coating creates a better integration of neural tissue than the smoother IrOx coating. The improved electrode‐tissue integration may reduce additional extracellular fluid buildup, which could shunt the injected current, requiring higher charge injection. In addition, we observed that microelectrode arrays with multiple shanks recruit a wider region of the cortex compared with single shank arrays. This may be associated with the larger implant footprint which severs additional axon, increasing network excitability evident in mild traumatic brain injury literature. Moreover, in vivo imaging of GCaMP response upon electrical stimulation was carried out at 24 h postelectrode implantation, allowing us to study the in vivo electrode performance without the interference of glial scarring. Furthermore, we investigated the effect of stimulation modality on neural activation. With voltage‐controlled stimulation, PC‐coated electrodes activate significantly more neural tissue than IrOx due to the lower impedance and the consequent higher injected current. With current‐controlled stimulation, we observed higher soma recruitment from PC sites than IrOx sites, likely due to its more intimate connection to neural tissue and increased nonuniform voltage field immediately nearby the electrode. Finally, for both materials, we observed more spatially distinct neuronal activation with shorter pulse widths. The findings of this work contribute to our understanding of cortical microstimulation using novel materials; using in vivo imaging in awake mice, we mimic conditions in human studies while directly visualized the region of activated neural tissue. Our results show that PC‐coated electrodes provide essential improvements in electrical stimulation applications in terms of increased energy efficiency compared with IrOx‐coated electrodes. Further work to assess the chronic in vivo stimulation performance of the PC electrode and other novel stimulation materials using this imaging setup is warranted.

## 5 Experimental Section

5.1

5.1.1

##### Electrode Modification

Two probe geometries were used in this study, both from NeuroNexus (Ann Arbor, MI), four‐shank iridium probes with four sites per shank (A4 × 4‐3 mm‐100‐125‐703; *n* = 5), and single‐shank iridium probes with 16 sites (A1 × 16‐3 mm‐50‐703; *n* = 5). Both probes were 3 mm long with a 703 μm^2^site area. The shank pitch for the four‐shank probes was 125 μm. Each electrode site was cleaned with isopropanol and rinsed with deionized water before electrode surface modification. Activation of iridium was done by delivering voltage‐controlled biphasic pulses from −0.8 to 0.9 V at a 50% duty cycle for 3200 s per site to maximize charge storage capacity. PC was prepared in 0.02 m EDOT and 2 mg mL^−1^ of acid functionalized CNTs using chronocoulometry using our published protocols.^[^ ^44^ ^]^

##### Electrochemical Characterization of Modified Electrodes

All in vitro measurements were carried out using a three‐electrode set up in PBS using Ag/AgCl as reference and Pt foil as the counter electrode. In vitro characterizations consisted of electrical impedance spectroscopy (EIS), CV, and CIL. EIS was measured by applying a 10 mV signal from 10 to 40 000 Hz. CV was measured to calculate the charge storage capacity and to identify the electrochemical signatures of the coated materials (−0.7 to 0.8 V at 1 V s^−1^ scan rate). CIL was carried out by delivering a biphasic asymmetric current‐based waveform (identical to the ones used for in vivo experiments in this work) and measuring the voltage excursion. From the voltage excursion, we can determine the charge density at which the *E*_mc_ (defined as the difference between the maximum cathodic voltage and the access voltage) exceeded −0.6 V for IrOx.^[^ ^18^ ^]^ The *E*_mc_ for PC was estimated to be −0.9 V based on CV.^[^ ^78^ ^]^ In vivo electrochemical characterization followed the same parameters as the in vitro set up except that a two‐electrode set up was used with the reference and counter electrodes shorted to a skull screw in the contralateral cortex. All electrochemical data were collected using the Autolab potentiostat.

##### Animal Surgery

All animal work was carried out under the guidelines of the University of Pittsburgh, Institutional Animal Care and Use Committee (IACUC protocol number: 21028691, PHS Assurance Number: D16‐00118). Ten male GCaMP6s mice, C57BL/6 J‐Tg (Thy1‐GCaMP6s) GP4.5Dkim/J (also known as GP4.3) mice were purchased from the Jackson Laboratory (Bar Harbor, ME). Animals were anesthetized with 75 mg kg^−1^ ketamine and 7.5 mg kg^−1^ xylazine cocktail for cranial window surgery and electrode implantation following aseptic procedures. The electrode arrays were sterilized by ethylene oxide gas 48 h before surgery. A reference screw was placed in the contralateral hemisphere and secured using UV‐curable dental cement (# 062066 Henry Shein). A high‐speed dental drill was used to remove the parietal bone over the somatosensory cortex. Electrodes were implanted at a 30° angle and inserted at a speed of 100–200 μm s^−1^ for 600 μm. Upon electrode implantation, a transparent silicone elastomer was used to seal the cranial window, covered by a 3 × 3mm^2^ glass coverslip. The electrode was dental cemented in place and the animal was allowed to recover and followed up with 3 days of analgesic and antibiotics.

##### Imaging and Electrical Stimulation

Images sensitive to GCaMP fluorescence over the exposed brain including the implanted electrode were acquired by wide‐field fluorescence imaging using a macroscope (MVX‐10, Olympus, Inc.) and high‐sensitivity camera (CoolSnap HQ2, Photometrics, Inc.) controlled by MetaMorph software. Time‐series images were acquired at 10 Hz. In addition, images of neurons expressing GCaMP6s around the implanted electrode were acquired by TPM (Ultima IV, Bruker Nano, Inc.) coupled to an ultrafast laser (Insight X3, Newport Spectra‐Physics, Inc.) using a 16× 0.8NA objective lens (Nikon, Inc.). The laser was tuned to 920 nm and time series were acquired with 1.27 × 1.27 μm pixel^−1^ resolution at 3 fps to capture GCaMP temporal responses.

Electrical stimulation was delivered using the Ripple GrapeVine system (Nano2+stim Ripple LLC, Salt Lake City, UT) via a 32 channel to 16 channel Omnetics adapter. The stimulation waveform was a charge‐balanced cathodic leading waveform (cathodic width: 100 μs; interphase delay: 100 μs; anodic phase: 200 μs). Stimulation was delivered at 50 Hz varying the amplitude of the cathodic phase from 5 to 60 μA over different trials in the same session. Each stimulation trial consisted of a 1 s ON period, and 3 s OFF period, repeated 6 times per electrode site. The energy for current‐controlled stimulation was calculated by integrating the absolute value of the product of voltage and current over a single pulse duration (300 μs ON period). For the delivery of a voltage‐controlled stimulus, an Autolab potentiostat (PGSTAT302N) was used in galvanostatic mode. The voltage pulse was biphasic with cathodic leading followed by an anodic phase twice the duration at half the amplitude, with the same pulse duration and trial repetition parameters. The electrical stimulus was synced to the beginning of both imaging methods using a National Instruments board (PCI‐6601, Austin, TX) to monitor the start‐of‐frame trigger from each system to deliver TTL pulses for each stimulation trial.

##### Image Analysis

All analyses were carried out in MATLAB. Wide‐field fluorescent time series were binned by a factor of 2 for a final resolution of 15 μm pixel^−1^ to increase the signal‐to‐noise ratio and reduce computational load. Quantification of GCaMP response to electrical stimulation was carried out on the mean of the six trials from each stimulation amplitude and stimulating electrode. Amplitude and extent of activation were extracted from the stimulation evoked responses. For this analysis, the images were cropped centered on the electrode for a final square region covering (1.2 × 1.2 mm^2^). The response amplitude (change in GCaMP fluorescence or Δ*F*/*F*_0_) was calculated by subtracting and dividing the mean of the initial 30 s baseline before electrical stimulation. *Z* scores were calculated by dividing by the standard deviation during this baseline period. For each stimulated trial, a 2D exponential decay function 1) was fitted to the maximum GCaMP response during the 1 s stimulation period. Where *A* is the amplitude of the background‐subtracted GCaMP response, **r** is a vector representing the space of the activated region, **r**_0_ represents the position of the center of the GCaMP response, and *w* is the radius of GCaMP response. We report the amplitude and extent (radius) of stimulation‐evoked GCaMP responses for those trials where the model explained at least 25% of the variance. In addition, for instances, where the GCaMP response amplitude is small or indifferent from noise, the algorithm ascribed near‐flat profiles to the data with artificially large radii (*w*). Considering that the model can reliably capture radii (*w*) up to 1/3 of the half‐width of the field of view, we labeled trials with GCaMP radii larger than 1/6 the width of the field‐of‐view as noise and excluded them from further analyses. Each statistical sample was reported as the mean of six trials for each electrode site.

(1) $$Y(r)=Ae^{-(r-r_{0})/w}$$

Neuronal expression of GCaMP across the mouse cortex is not homogeneous.^[^ ^79^ ^]^ To enable comparison between PC and IrOx in nearby but different depth locations, we normalized the GCaMP intensity and radius from PC sites to their immediate distal IrOx sites for linear arrays. For four‐shank arrays, the normalization was carried out by taking the ratio of PC sites to their parallel IrOx sites in the same position. For example, in 4 × 4 arrangement where sites (1,1) and (1,3) are coated with PC and sites (1,2) and (1,4) are coated with IrOx, the normalizations were (1,1)/(1,3) and (1,2)/(1,4).

The two‐photon imaging was analyzed to quantify the number and location of electrically activated somas using custom MATLAB algorithms. The time‐series images were corrected for motion before analysis. Change in fluorescence was calculated by subtracting and dividing the 30 s of the baseline period before electrical stimulation. Neuronal somas were segmented from the average time‐series image based on a local intensity threshold. Low‐frequency intensity variations were removed from the average image using a high‐pass filter to help identify and segment GCAMP‐expressing neuronal soma. Electrically activated neurons were determined by correlating the stimulus temporal waveform with the time series from segmented neuronal ROIs resulting in an *r* score (*r*), which were transformed to *t* scores to evaluate significance (one‐sided *t*‐test *p < 0.05*).^[^ ^80^ ^]^ We Bonferroni corrected the *p‐value* by the total number of ROIs to account for multiple comparisons. Neuropil fluorescence consists of the dense network of dendrites and synapses and out‐of‐plane cell bodies within the field of view. We quantified neuropil fluorescence by subtracting the segmented somas from the time series.

Maps of the threshold current for activating neural elements near the electrode were generated by first obtaining a binary map of pixels that are significantly activated by electrical stimulation and labeling the activated pixels by the lowest current amplitude at which they are activated. To obtain the change in threshold current as a function of distance away from the electrode center, the images were binned with concentric circles in intervals of 15 μm per bin. We define the average spatial selectivity (μA μm^−1^) as the slope of threshold current versus distance away from the center of the electrode for each pulse width.

##### Finite Element Modeling

To study the impact of potential differences in the electric field imparted by current delivery through IrOx and PC sites, a finite element model was established in the AC/DC module in COMSOL v5.5. The 2D model consists of a working electrode (WE) and a reference electrode (RE) suspended in brain tissue. The conductivity and relative permittivity of IrOx, PC, and the brain tissue is shown in **Table** **2**. As an estimation, we chose literature values for the material properties for the modeling study. The IrOx thin‐film conductivity was calculated by taking the inverse of the resistivity described in the study by Yuxue Liu and Goto^[^ ^81^ ^]^ at 1 kHz and room temperature. The relative permittivity was obtained from the study by Dias et al.,^[^ ^82^ ^]^ which reported a design and characterization of noninvasive iridium oxide biopotential electrodes. The electrical conductivity of PC was obtained from the study by Liu et al.^[^ ^83^ ^]^ where they evaluated the thermoelectric properties at room temperature and 50 °C for composites of PEDOT:polystyrene sulfonate/multi walled carbon nanotubes/graphene.^[^ ^84^ ^]^ We obtained the value at room temperature. Finally, the relative permittivity for PC was obtained from the study by Sanad et al.^[^ ^84^ ^]^ where these values were used to simulate the thermoelectric behavior of a CNT‐based harvester, and simulation results were experimentally validated to be in acceptable agreement with simulation.

**Table 2:** Material properties used for finite element modeling

	Reference Screw (SS)	IrOx	PC	Brain
Electrical Conductivity [S m^−1^]	4.032e6	2.8e6^[^ ^81^ ^]^	0.026^[^ ^83^ ^]^	1.5e−1^[^ ^86^ ^]^
Relative Permittivity	1	3.5e5^[^ ^82^ ^]^	3.05^[^ ^84^ ^]^	2.5e3^[^ ^86^ ^]^

IrOx sites were represented with a circular disk of 703 μm^2^. To model the roughness imparted by the nanofibrous topography of PC, these sites were represented with a randomly generated polar curve with random roughness following a Gaussian distribution. This distribution was chosen to mimic the smooth but random variations observed in SEM images from these sites. The roughness of the electrode model was controlled by changing the spatial frequency and spectral exponent. We estimated the spatial resolution and spectral exponent of PC by deriving values obtained from SEM in ImageJ (Figure S1, S2a,b, S3, Supporting Information). Specifically, the spatial resolution was obtained by dividing the number of peaks in the 1D profile by the total distance (μm^−1^). The spectral exponent was estimated by plotting the power spectrum density of the 1D profile and finding the slope of the linear region that is within the relevant wavenumber range for the smallest feature of the PC composite, i.e., CNTs.^[^ ^85^ ^]^ From the PC coating in Figure S3, Supporting Information, we estimated a spectral resolution of 76 μm^−1^ and a spectral exponent of 1.5.

The voltage profile as a function of distance from the edge of the electrode was fitted to an exponential two‐phase decay (Equation ((2), (3))–(4)) in GraphPad PRISM, using least squares regression, where *V*_peak_ represents the amplitude of the voltage field when the distance is 0 μm (*X* = 0). *V*_0_ represents the amplitude of the voltage field when the distance approaches 100 μm (*X* = 100). *K*_fast_ and *K*_slow_ represent the two rate constants for the slow and the fast decay phases. *f*_fast_ is the fraction of the amplitude (from *V*_peak_ to *V*_o_) accounted for by the faster of the two components.

(2) $$V_{\text{slow}}=(V_{\text{peak}}-V_{0})\times (1-f_{\text{fast}})$$

(3) $$V_{\text{fast}}=(V_{\text{peak}}-V_{0})\times f_{\text{fast}}$$

(4) $$Y(x)=V_{\text{slow}}\exp(-k_{\text{slow}}X)+V_{\text{fast}}\exp(-k_{\text{fast}}x)+V_{0}$$

##### Statistical Analysis

Preprocessing of imaging data is described in detail in Section 6.5. Statistical analyses were carried out using GraphPad Prism 8.0. Two‐way ANOVA with Sidak posthoc analysis was used for the following statistical comparisons: 1) the effect of electrode material on GCaMP intensity and radius as a function of increasing stimulation current amplitude, 2) the effect of electrode geometry on GCaMP intensity and radius as a function of increasing stimulation current amplitude, and 3) the effect of electrode material on activated neuron soma and neuropil intensity for different stimulation pulse widths. A *p*‐value of less than 0.05 was deemed significant. A Wilcoxon signed‐rank test was used for testing the effect of electrode material on normalized GCaMP intensity and radius. A two‐tailed student *t*‐test was used for testing the effect of electrode material on activated soma and neuropil intensity for voltage‐controlled and current‐controlled stimulations. A *p*‐value of less than 0.05 was deemed significant. Linear regression was used to investigate the relationship between the average spatial selectivity and pulse width, a *p*‐value of less than 0.05 for the slope was deemed significant. All sample sizes were denoted in the captions of respective figures. Each statistical sample was a mean of six trials for each electrode site. All error bars represented standard error of the mean.

## Conflict of Interest

The authors declare no conflict of interest.

## Data Availability Statement

The data that support the findings of this study are available from the corresponding author upon reasonable request.

## Supporting information

Supplementary Material
