# Supplementary material for: Imaging the Efficiency of Poly(3,4‐ethylenedioxythiophene) Doped with Acid‐Functionalized Carbon Nanotube and Iridium Oxide Electrode Coatings for Microstimulation
Source: Adv Nanobiomed Res. 2021 May 3;1(7):2000092. doi: 10.1002/anbr.202000092 (PMC8552016; doi:10.1002/anbr.202000092)
Supplement: Supplementary file 1 — Supplementary Material [file ANBR-1-2000092-s001.pdf]

## Supporting Information

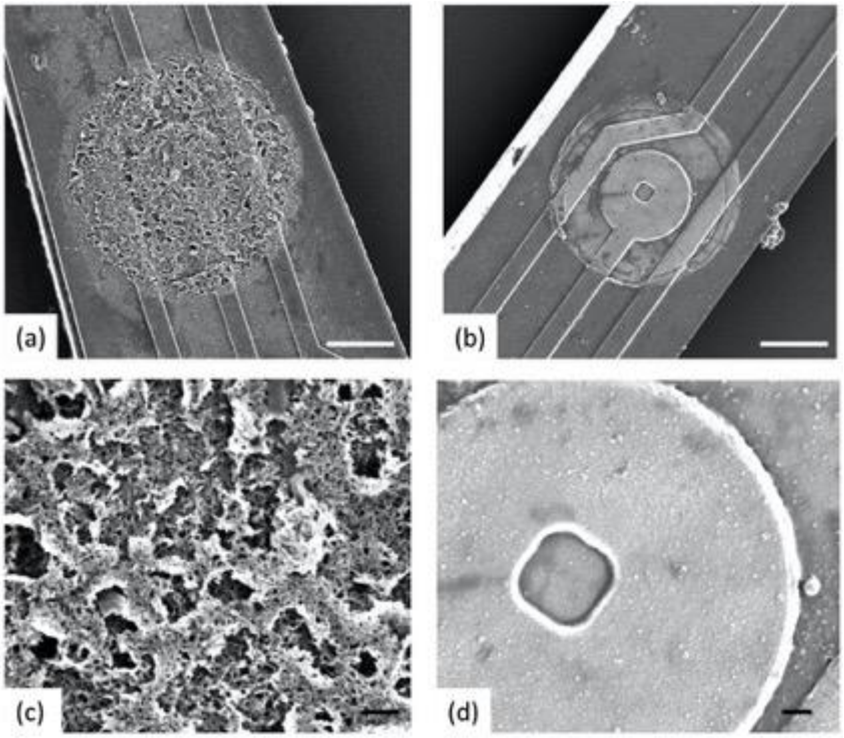


**Figure S1. Scanning electron microscopy of acutely explanted Michigan arrays with PEDOT/CNT and IrOx sites.**

**Sites were modified with (a) PEDOT/CNT and (b) IrOx thinfilm. Scale bars represent 10 μm. (c) Magnified view of a PEDOT/CNT coated surface. Scale bar represent 1 μm. (d) Magnified view of a IrOx thinfilm surface. Scale bar represent 1 μm.**

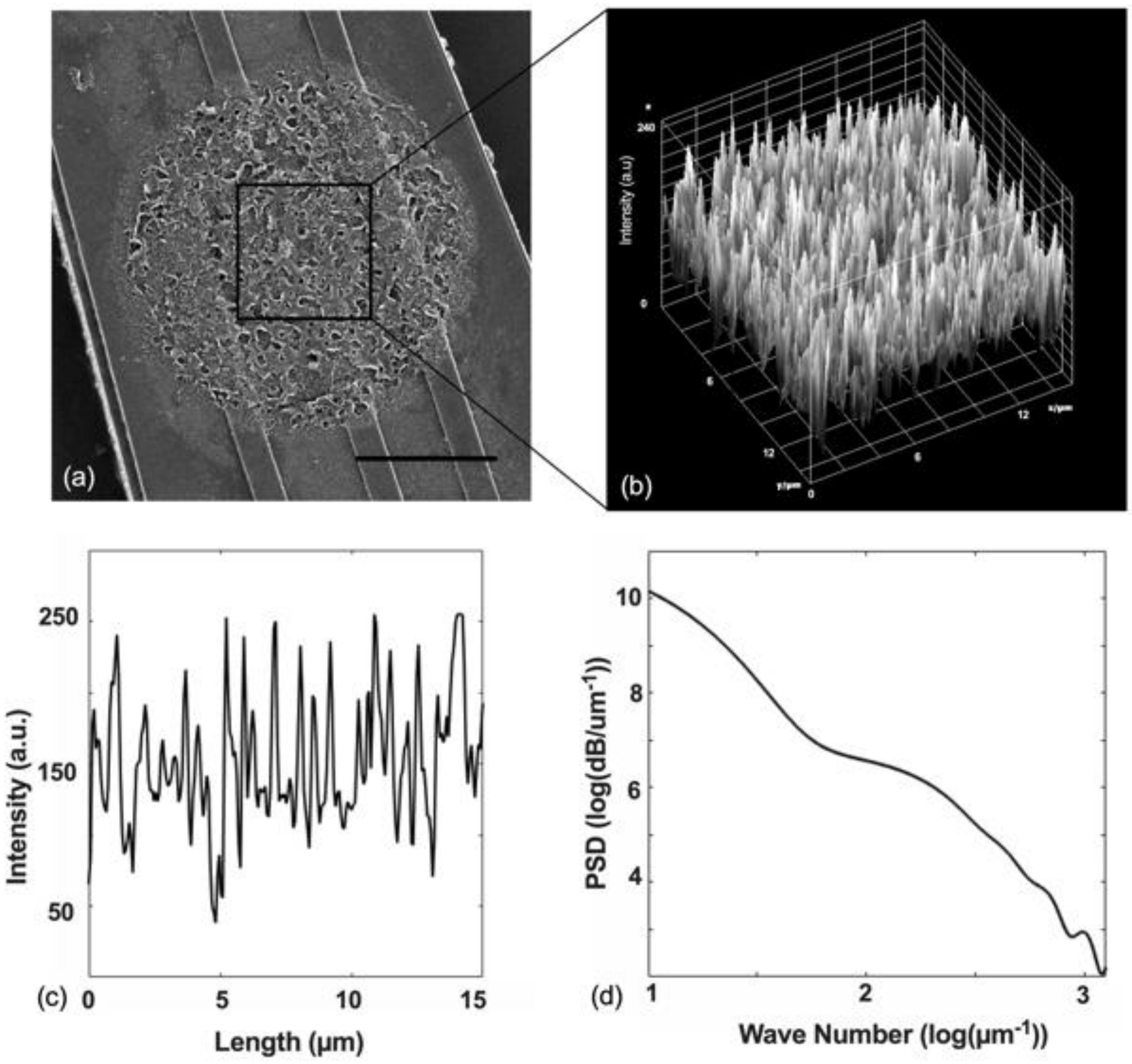


**Figure S2. Estimation of fractal features of the PEDOT/CNT surface.**

**(a) SEM of a PEDOT/CNT coating on an iridium substrate. Black box encompass a 3D visualization of surface topography in (b). (b) 3D representation of surface topography based on SEM intensity. (c) 1D profile of the PEDOT/CNT surface topography. (d) 1D profile of the PEDOT/CNT surface topography in the Fourier domain.**

**Table S1. Parameters describing the two-phase exponential decay function representing voltage field distribution nearby PC and IrOx electrodes.**

| | $V_{peak}$ | $V_0$(V/m) | $K_{fast}$ | $K_{slow}$ | $V_{slow}$(V/m) | $V_{fast}$ (V/m) | r (μm) | R-squared |
|---|---|---|---|---|---|---|---|---|
| PC | 3.9e5 | 4.7e4 | 0.19 | 0.036 | 2.4e5 | 1.08e5 | 32.5 | 0.99 |
| IrOx | 3.5e5 | 3.9e4 | 0.14 | 0.030 | 1.9e5 | 1.1e5 | 39.9 | 0.99 |
| % difference | 11.4 | 21 | 35.7 | 20 | 26 | 2.6 | 18.5 | - |

## 1.1.1 Explanted arrays

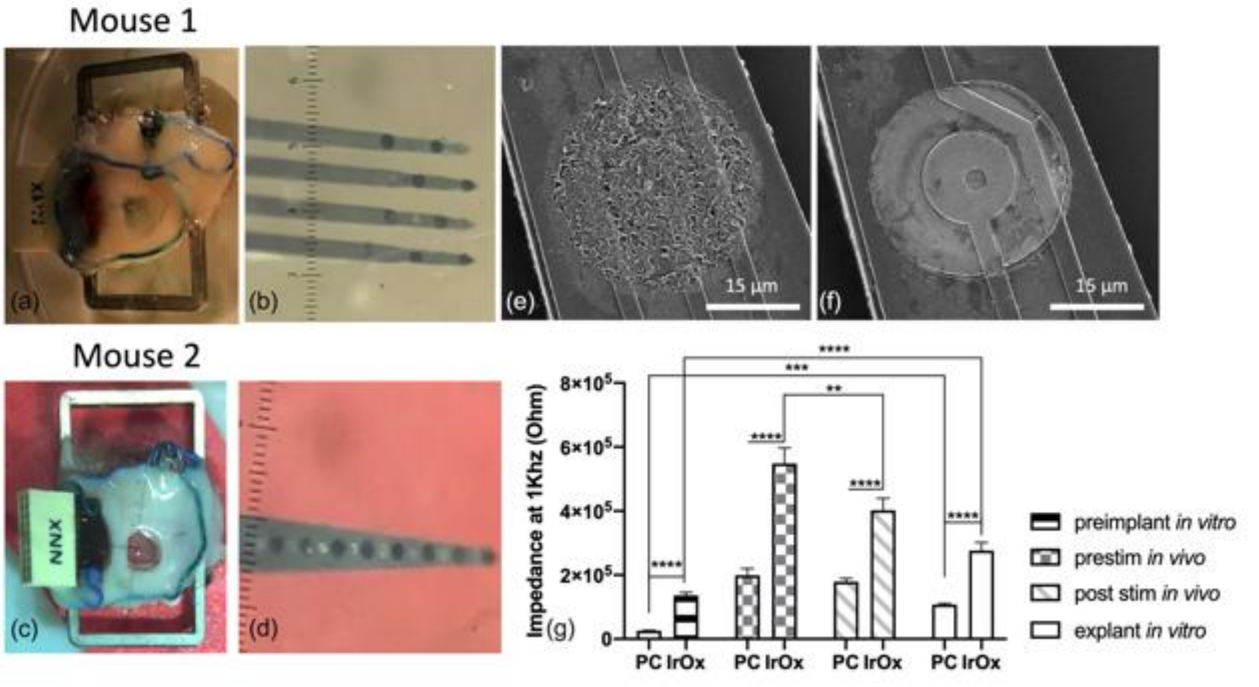


**Figure S3. Explant examination. (a) Explanted skull showing headframe, cranial window, a 4x4 electrode array and reference screw. (b) Zoomed in view of the 4x4 electrode array. (c) Explanted skull from a different mouse showing the same components of mouse 1 but a linear array. (e) SEM of a representative array showing an electrode site coated with PEDOT/CNT. (f) SEM of a representative array showing an electrode site with IrOx thinfilm. (g) Impedance at 1kHz of explanted arrays coated with PC and IrOX pre-implant**

**and explanted. There was no significant difference in impedance between pre and post stimulation measurements. (h). Charge injection limit for PC and IrOx pre and post implantation. Measurements were performed in PBS with a three electrode setup against Ag/AgCl reference electrode. N = 16 electrode sites for IrOx and PC, respectively. Two-way ANOVA, Tukey's post hoc correction. *** $p < 0.001$, **** $p < 0.0001$.**
